# Trends in alcohol and tobacco use among Brazilian students: 1989 to 2010

**DOI:** 10.1590/S0034-8910.2015049005860

**Published:** 2015-10-02

**Authors:** Zila M Sanchez, Mariangela Cainelli Oliveira Prado, Adriana Sanudo, Elisaldo A Carlini, Solange A Nappo, Silvia S Martins

**Affiliations:** I Centro Brasileiro de Informações sobre Drogas Psicotrópicas. Departamento de Medicina Preventiva. Universidade Federal de São Paulo. São Paulo, SP, Brasil; II Área de Bioestatística. Departamento de Medicina Preventiva. Universidade Federal de São Paulo. São Paulo, SP, Brasil; III Department of Epidemiology. Columbia University. New York, NY, USA

**Keywords:** Alcohol Drinking, trends, Smoking, trends, Students, Adolescent Behavior

## Abstract

**OBJECTIVE:**

To analyze temporal trends of the prevalence of alcohol and tobacco use among Brazilian students.

**METHODS:**

We analyzed data published between 1989 and 2010 from five epidemiological surveys on students from the 6^th^ to the 12^th^ grade of public schools from the ten largest state capitals of Brazil. The total sample consisted of 104,104 students and data were collected in classrooms. The same collection tool – a World Health Organization self-reporting questionnaire – and sampling and weighting procedures were used in the five surveys. The Chi-square test for trend was used to compare the prevalence from different years.

**RESULTS:**

The prevalence of alcohol and tobacco use varied among the years and cities studied. Alcohol consumption decreased in the 10 state capitals (p < 0.001) throughout 21 years. Tobacco use also decreased significantly in eight cities (p < 0.001). The highest prevalence of alcohol use was found in the Southeast region in 1993 (72.8%, in Belo Horizonte) and the lowest one in Belem (30.6%) in 2010. The highest past-year prevalence of tobacco use was found in the South region in 1997 (28.0%, in Curitiba) and the lowest one in the Southeast in 2010 (7.8%, in Sao Paulo).

**CONCLUSIONS:**

The decreasing trend in the prevalence of tobacco and alcohol use among students detected all over the Country can be related to the successful and comprehensive Brazilian antitobacco and antialcohol policies. Despite these results, the past-year prevalence of alcohol consumption in the past year remained high in all Brazilian regions.

## Abstract (pt)

**OBJETIVO:**

Analisar a tendência temporal da prevalência de uso de álcool e tabaco por estudantes brasileiros.

**MÉTODOS:**

Foram analisados, entre 1989 e 2010, dados de cinco inquéritos epidemiológicos com estudantes do sexto ano do ensino fundamental ao terceiro do ensino médio, matriculados em escolas públicas nas 10 maiores capitais do Brasil. A amostra foi de 104.104 estudantes e os dados foram coletados em sala de aula. O instrumento de coleta – questionário de autopreenchimento da Organização Mundial da Saúde – e os procedimentos de amostragem e ponderação foram os mesmos nos cinco inquéritos. Teste de Qui-quadrado de tendências foi utilizado para comparar as prevalências entre os anos.

**RESULTADOS:**

A prevalência de uso de álcool e tabaco variou nos anos e entre as capitais estudadas. O uso de álcool diminuiu em todas as 10 capitais (p < 0,001), em 21 anos. Houve também diminuição significativa do uso de tabaco em oito capitais (p < 0,001). A maior prevalência de uso de álcool foi observada na região Sudeste, em 1993 (72,8% em Belo Horizonte), e a menor, em Belém (30,6%), em 2010. A maior prevalência de uso do tabaco no último ano foi na região Sul, em 1997 (28,0% em Curitiba) e a menor na região Sudeste, em 2010 (7,8% em São Paulo).

**CONCLUSÕES:**

A tendência de queda na prevalência de tabagismo e uso de álcool entre estudantes, observada em todo o País, pode estar relacionada com políticas antitabaco e antiálcool bem-sucedidas e abrangentes. Apesar destes resultados, a prevalência de uso de álcool no último ano continuou elevada em todas as regiões brasileiras.

## INTRODUCTION

Alcohol and tobacco are the drugs most widely used by adolescents worldwide and responsible for a high global burden of disease in several countries.^9^ Drinking and smoking during adolescence are associated with many of the major causes of preventable morbidity and mortality and may be addressed using focused public health interventions.^4^ ^,^ ^15^ In Brazil, while the media and the government are focused on a potential large national “epidemic” of crack cocaine, legal drugs such as alcohol and tobacco are still the most prevalent among middle and high schools students. For example, 0.6% of them reported lifetime use of crack cocaine *versus* 60.5% and 16.9% lifetime alcohol and tobacco use, respectively.^22^

Trends for alcohol use among adolescents diverge between countries and continents. For instance, in Russia, from 1994 to 2011, weekly drinking reported by 15-year-old adolescents increased from 13.0% to 28.0% among boys and from 6.0% to 15.0% among girls.^29^ However, opposite trends have been reported by 13- to 17-year-old students in New Zealand, where past-month binge drinking dropped from 44.0% to 20.0% from 2001 to 2012.^6^ In the past two decades, the lifetime prevalence of alcohol use among high school students in Canada,^8^ Japan^23^ and the United States^16^ decreased from 20.0% to 50.0%. In the Netherlands, stabilization in alcohol use was detected in the 1990s, followed by an increase in the first decade of the 2000s, from a lifetime prevalence of alcohol use of 68.6% in 1993 to 88.4% in 2000,^24^ attributed to the arrival of alcopops in the country.^27^ In Latin America, countries have less regular data and, consequently, trends over time cannot always be examined. According to data from the *Organización de los Estados Americanos,* the prevalence of recent use of alcohol (past 30 days) in Paraguay, Chile, and Ecuador was 40.0% among high school students between 2003 and 2010.^a^ In Chile, the prevalence of past-year alcohol use was high and relatively stable (61.0% to 63.0%) from 2001 to 2013.^b^ In Mexico, from 2006 to 2009, the prevalence of past-year alcohol use was 50.0%.^30^

Tobacco use among students shows a worldwide trend of decrease since 2000, varying according to country, subjects, and local public policies. The Global Young Tobacco Survey^31^ shows that current smoking prevalence varies from 30.0% in some Eastern European countries (Latvia, Lithuania and Bulgaria) to 1.0% in Asian countries such as China, Vietnam and Pakistan. In Europe, the different stages of the tobacco epidemic among countries complicate the establishment of a general trend. However, the lifetime prevalence of smoking was stable between 1995 and 2001 and started to decline in the 2000s. Iceland is the only country where smoking in the past 30 days has been falling over the whole time period of 1995-2011, from 32.0% in 1995 to 10.0% in 2011.^14^ In the United States, prevalence increased during the 1970s, stabilized in the 1980s and 1990s and decreased in the 2000s, reaching now the lowest historical prevalence.^16^ As a public health concern, Latin American cities still have high rates of tobacco use among 13- to 15-year-olds, varying from 34.0% of daily tobacco smoking in Santiago (Chile) and Bogota (Colombia) to 12.0% in Rio de Janeiro (Brazil).^31^ In addition, within a country it is possible to find diverse trends per state.^1^

Brazil, the sixth world economy,^c^ lacks data about long-term trends in alcohol and tobacco use by adolescents. Target-oriented prevention among adolescents requires consecutive cross-sectional epidemiologic surveys of alcohol and tobacco use, which enables trends analysis over time. These trends allow us to anticipate the frequency of users in the next years and the potentially predictable social costs of abuse or dependence to the community.^8^ The usual limitation of trend analysis is that, several times, the instrument used, sampling design, and data analysis are not the same between surveys, thus limiting data comparability. Since 1987, the Brazilian Center of Information on Psychotropic Drugs (CEBRID) collects data on drug use among Brazilian students using the same study design, instrument, and central coordination team, which is essential for a comparison of two decades.^12^

This study aimed at analyzing temporal trends of the prevalence of alcohol and tobacco use among Brazilian students, from 1989 to 2010.

## METHODS

Data came from five cross-sectional surveys of public school-attending youths in the 10 largest state capitals of Brazil, with survey data collected in classrooms in 1989, 1993, 1997, 2004 and 2010 from a total sample of 104,104 students. In each year, the target population was a representative sample of middle and high school students (6^th^ to 12^th^ grade), with a two-step random selection process. The 10 state capitals represented the five geoeconomic regions of Brazil, which differ culturally, economically, socially, climatically and racially from each other. The capitals and the regions included in all the surveys were: Porto Alegre and Curitiba, in the South region; Sao Paulo, Belo Horizonte and Rio de Janeiro, in the Southeast; Belem, in the North; Fortaleza, Recife and Salvador, in the Northeast; and Brasilia, in the Midwest. However, in the 2004 and 2010 surveys, all the Brazilian capitals were included in the sample to allow comparative data with the other years; we included only the 10 capitals for which data are available in all years surveyed for the trend analysis.

Sampling was clustered (by school) and stratified (by socioeconomic characteristics) into two stages, first by school and then by class, as proposed by Kish.^17^ For each survey, data were weighted for school selection, classroom selection and nonresponse. All the students in a selected classroom were asked to answer the survey and the gathered students’ response rate was always higher than 90.0% in each survey.

Sample sizes were 19,002 in 1989, 24,634 in 1993, 15,501 in 1997, 21,712 in 2004, and 23,255 in 2010. A larger number of female students were found in all four surveys (1.0% to 2.0% higher than the proportion of boys), which is in line with the proportions recorded by the census in Brazil.^d^ The mean age of the sample was 13 years in all surveys (from 13.2 to 13.7), with the standard deviation (SD) varying between surveys (from 0.4 to 0.8).

Each survey of this study was approved by the Universidade Federal de São Paulo (UNIFESP) Research Ethics Committee (Process 0348/08).

Data were gathered by trained interviewers in the classrooms, in the absence of teachers. A questionnaire with closed-ended questions based on the standardized World Health Organization (WHO) items and adapted for use in Brazil by Carlini-Cotrim et al^e^ was used for the five surveys in the same cities. The outcome measures (alcohol and tobacco use) were never changed throughout the years.

The questionnaire collected sociodemographic data (sex, age, school grade, socioeconomic level), frequency of school attendance, use of nonprescription psychotropic drugs (anxiolytics, amphetamines, anticholinergics, barbiturates, codeine-based cough syrups, and opiate analgesics), use of alcohol, tobacco and illegal drugs (inhalants, marijuana and cocaine). A fictitious drug was included in the questionnaire to test for authenticity.

The outcome variables for the trend analysis were past-year alcohol and tobacco use. The question for past-year use of alcohol was: “In the past year, i.e., the last 12 months, have you had any alcoholic beverage?” (yes or no). For tobacco use: “In the past year, i.e., the last 12 months, have you smoked any tobacco cigarette?” (yes or no).

Data were analyzed per city and presented in the graphs per Brazilian region, according to the characteristics described in the sampling selection.

Each dataset was double entered by different typists, allowing correction for typing errors. Since all questions contained several items, split-half reliability testing was applied to eliminate inconsistencies such as answering no to item “a” (lifetime use) and yes to item “b” (past-year use). All questionnaires containing affirmative responses for the fictitious drug, or containing more than three invalidated or blank responses were excluded from the dataset. On average, 20,000 students answered the questionnaire per year and about 2.0% were excluded from each survey.

Prevalence analyses were conducted on data weighted to correct unequal probabilities of selection into the sample. The complex survey design considered the school the primary sampling unit, the expansion weight to represent the population of Brazilian students, and the final probability of drawing students who answered the questionnaire. Analyses were performed using Stata Version 11. Results are presented via weighted proportions (wg%) and 95% confidence interval (95%CI). Trends between surveys were analyzed by the Chi-square test for trends (c^2^ trend test) with a 5% level of significance. This test of proportions is used if the table of data has two columns and three or more rows and determines whether there is a linear trend between row (or column) number and the fraction of subjects in the left column (or top row). The Bonferroni correction was used for multiple comparisons between surveys.

## RESULTS

The prevalence of past-year alcohol use varied between years and cities. During the period, the highest prevalence of alcohol use was found in the Southeast and South region in 1993 (72.8% in Belo Horizonte and 71.4% in Curitiba), and the lowest in the North (30.6% in Belem) in 2010. The year of lowest prevalence was 2010 for the 10 capitals.

**Figure 1:**
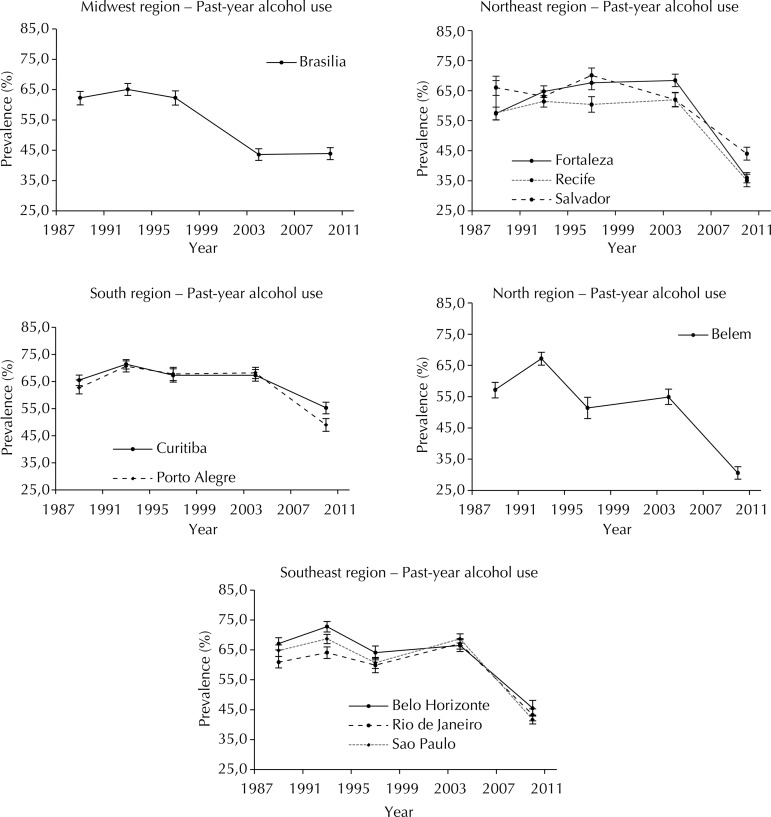
Trends in the prevalence of past-year alcohol use by Brazilian students in 1989, 1993, 1997, 2004, and 2010.

Chi-square trend test results showed a significant decrease in alcohol past-year use in all 10 capitals (p < 0.001) in the 21-year (1989 to 2010) comparison (Table 1). Belem showed the highest decrease, with a rate change from 62.6% (95%CI 60.5;64.6) in 1993 to 30.6% (95%CI 28.6;32.6) in 2010 and a prevalence ratio of 0.49. In general, the highest decrease in alcohol use occurred between 2004 and 2010, except in Brasilia, the Federal District of Brazil, where the main decrease happened between 1997 and 2004, with stabilization in the last survey (2010). Also, the highest consumption of alcohol by adolescent students in 2010 occurred in Curitiba (55.3%, 95%CI 53.1;57.4) (Figure 1).

**Table 1:** Trends in past-year alcohol use by Brazilian students, according to number of cases, weighted proportions (wg%) and 95%CI.

Region	City	1989			1993			1997			2004			2010			
		n	wg%	95%CI	n	wg%	95%CI	n	wg%	95%CI	n	wg%	95%CI	n	wg%	95%CI	p*
Midwest	Brasilia	1,873	62.3	60.0;64.4	2,208	65.1	63.1;67.0	1,577	62.3	59.9;64.6	2,637	43.6	41.7;45.5	2,425	43.9	41.9;45.9	< 0.001
Northeast	Fortaleza	1,987	57.4	55.2;59.5	2,251	64.7	62.7;66.6	1,651	67.6	65.3;69.8	1,870	68.4	66.3;70.5	2,977	36.0	34.3;37.7	< 0.001
	Recife	1,833	57.6	55.3;59.8	2,541	61.4	59.5;63.3	1,366	60.4	57.8;63.0	1,692	62.0	59.7;64.3	2,033	35.1	33.0;37.2	< 0.001
	Salvador	1,384	66.0	63.4;68.4	2,829	63.1	61.3;64.9	1,269	70.1	67.5;72.5	1,570	62.0	59.6;64.4	2,091	44.0	41.9;46.2	< 0.001
North	Belem	1,494	57.2	54.6;59.6	2,123	62.6	60.5;64.6	829	51.4	48.0;54.8	1,558	54.9	52.5;57.4	2,067	30.6	28.6;32.6	< 0.001
Southeast	Belo Horizonte	1,998	67.1	65.0;69.1	2,433	72.8	71.0;74.5	1,661	64.1	61.7;66.3	2,230	66.5	64.5;68.4	1,427	45.5	42.9;48.1	< 0.001
	Rio de Janeiro	2,521	60.9	59.0;62.8	2,303	64.1	62.1;66.0	1,614	59.9	57.4;62.2	2,758	67.1	65.3;68.8	2,347	43.3	41.3;45.3	< 0.001
	Sao Paulo	2,384	64.8	62.8;66.7	3,555	68.7	67.1;70.2	2,730	60.7	58.8;62.5	3,522	68.9	67.3;70.4	4,073	41.7	40.2;43.2	< 0.001
South	Curitiba	2,224	65.5	63.5;67.4	2,513	71.4	69.6;73.1	1,430	67.3	64.8;69.8	1,823	67.3	65.1;69.4	2,090	55.3	53.1;57.4	< 0.001
	Porto Alegre	1,304	62.7	60.0;65.2	1,878	70.6	68.5;72.6	1,374	67.8	65.3;70.2	2,052	68.2	66.1;70.2	1,725	49.0	46.6;51.3	< 0.001

* c^2^: trend test.

The prevalence of past-year tobacco smoking was lower than past-year alcohol use in all of the cities and in the five surveys, varying between years and cities. During the period, the highest prevalence occurred in the South region in 1997 (28.0%, in Curitiba) and the lowest in the Southeast in 2010 (7.8%, in Sao Paulo).

**Figure 2:**
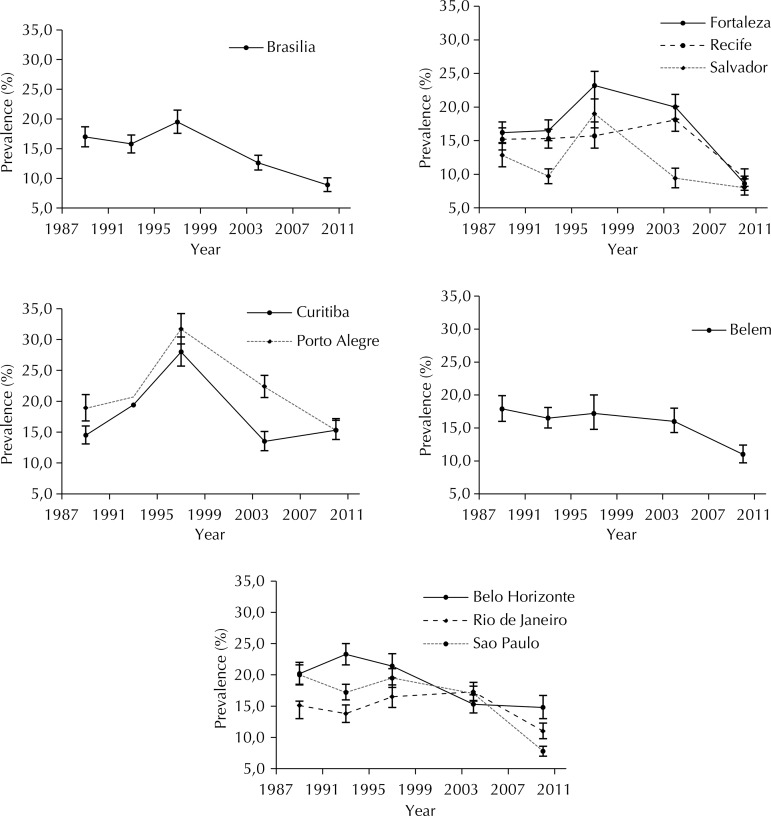
Trends in the prevalence of past-year tobacco use by Brazilian students in 1989, 1993, 1997, 2004, and 2010.

In the 21-year (1989 to 2010) comparison, the Chi-square trend test showed a significant decrease in tobacco past-year use in students in eight capitals (p < 0.001), the exceptions being Rio de Janeiro and Curitiba (Table 2). Nine cities showed a trend for decrease from 1997 to 2010, and the only increase in the last survey was found in Curitiba (from 13.5% to 15.3%). The highest decrease was found in Fortaleza, with prevalence changing from 23.2% (95%CI 21.2;25.3) in 1993 to 8.6% (95%CI 7.6;9.7) in 2010 and a prevalence ratio of 0.37. In general, tobacco use decreased the most between 1997 and 2010, except for Recife and Rio, where it decreased later, from 2004 to 2010. Also, tobacco was most consumed by adolescent students in 2010 in the South region (Figure 2).

**Table 2:** Trends in past-year tobacco use by Brazilian students in 1989, 1993, 1997, 2004, and 2010, according to number of cases, weighted proportions (wg%) and 95%CI.

Region	City	1989			1993			1997			2004			2010			
		n	wg%	95%CI	n	wg%	95%CI	n	wg%	95%CI	n	wg%	95%CI	n	wg%	95%CI	p*
Midwest	Brasilia	1,873	17.0	15.3;18.7	2,208	15.8	14.3;17.3	1,577	19.5	17.6;21.5	2,637	12.6	11.4;13.9	2,425	8.9	7.8;10.1	< 0.001
Northeast	Fortaleza	1,987	16.2	14.6;17.8	2,251	16.5	15.0;18.1	1,651	23.2	21.2;25.3	1,870	20.0	18.2;21.9	2,977	8.6	7.6;9.7	< 0.001
	Recife	1,833	15.2	13.6;16.9	2,541	15.3	13.9;16.7	1,366	15.7	13.9;17.8	1,692	18.1	16.4;20.1	2,033	9.4	8.2;10.8	< 0.001
	Salvador	1,384	12.8	11.1;14.7	2,829	9.7	8.6;10.8	1,269	19.0	16.9;21.2	1,570	9.4	8.0;10.9	2,091	8.0	6.9;9.3	< 0.001
North	Belem	1,494	17.9	16.0;19.9	2,123	16.5	15.0;18.1	829	17.2	14.8;20.0	1,558	16.0	14.3;18.0	2,067	11.0	9.7;12.4	< 0.001
Southeast	Belo Horizonte	1,998	20.2	18.5;22.0	2,433	23.3	21.6;25.0	1,661	21.4	19.5;23.4	2,230	15.3	13.9;16.8	1,427	14.8	13.0;16.7	< 0.001
	Rio de Janeiro	2,521	15.1	13.7;16.5	2,303	13.8	12.4;15.2	1,614	16.5	14.8;18.4	2,758	17.3	15.9;18.8	2,347	11.0	9.8;12.3	0.072
	Sao Paulo	2,384	20.0	18.4;21.6	3,555	17.2	16.0;18.5	2,730	19.5	18.0;21.0	3,522	17.0	15.8;18.2	4,073	7.8	7.0;8.6	< 0.001
South	Curitiba	2,224	14.5	13.1;16.0	2,513	19.4	17.9;21.0	1,430	28.0	25.7;30.4	1,823	13.5	12.0;15.1	2,090	15.3	13.8;16.9	0.172
	Porto Alegre	1,304	18.9	16.8;21.1	1,878	20.7	18.9;22.6	1,374	31.7	29.3;34.2	2,052	22.4	20.6;24.2	1,725	15.4	13.8;17.2	0.038

* c^2^: trend test..

## DISCUSSION

The past-year use of alcohol among adolescents in Brazil between 1989 and 2010 declined from 62.2% to 42.0%, while tobacco use declined from 16.8% to 10.4%. Despite the prevalence decrease in these 21 years, alcohol use was more prevalent than tobacco use in all 10 cities and all time points. The relative tobacco reduction between 1989 and 2010 ranged from 18.5% in Porto Alegre to 61.0% in Sao Paulo. Concerning the prevalence of alcohol use, we observed a relative reduction from 1989 to 2010 in all 10 cities, ranging from 15.6% in Curitiba to 46.5% in Belem.

The estimated smoking prevalence in 2010 was lower than in 1989 in nine cities, Curitiba being the exception. One economic reason should be considered a substantial contributor to the difference in the trends in Curitiba: Brazil is the second largest tobacco producer worldwide, with 97.0% of the national production in its Southern region (where Curitiba is located). In the South, nearly 62.0% of the municipalities have their economies almost entirely dependent on the tobacco industry.^f^

Curitiba houses the second largest tobacco company in Brazil, Philip Morris, which is the employer of 3,000 citizens. Although the interaction between the effectiveness of drug control policies and context-level factors is an important consideration in complex interventions,^11^ the economic dependence of a community on the tobacco industry has not been examined in the scientific literature. As civil society advocacy is one of the most important elements for tobacco control success,^3^ it is not hard to suppose the inherent conflict of interest between economic concerns and social responsibility activities.

Apart from the Curitiba scenario, this historical decrease in the prevalence of tobacco smoking seems to be strongly influenced by the Brazilian national campaigns against tobacco use.^7^ ^,^ ^12^ ^,^ ^21^ Since 1986, the Brazilian government issued several national laws on tobacco control, including restricting cigarette advertising and consumption in closed public places, warnings about cigarette smoking harms on the packs, and legislative constraints for outlets selling tobacco products.^21^ In addition to those population-based preventive approaches in tobacco control policies, in 2004 a risk factor-based intervention, grounded on nicotine-free dependence treatment, was created by the Brazilian Unified Health System (SUS), and offered for free to any citizen. Since 2005, the Brazilian tobacco control program was decentralized and the most significant achievements occurred at the state level, depending on the political commitment at local and regional levels.^7^ The Brazilian pattern of decreasing tobacco use prevalence, with a floating decrease in prevalence from 1987 to 2004 and a steep decline from 2004 to 2010, presents valuable insights regarding tobacco prevention and control. This suggests that centralized and broad population-based policies based on antismoking regulations can decrease tobacco smoking prevalence effectively.^19^ However, in countries with large socioeconomic and cultural disparities as Brazil, decentralizing the program coordination may be relevant, using capacity building at local and regional levels for adopting risk factor-based interventions towards socially defined population subgroups at increased risk.

Regarding alcohol use, we observed a surprising decrease in past-year prevalence. Alcohol is the most prevalent drug in Brazil for all age groups^g^ and is regulated by laws that are not usually enforced.^2^ For example, although federal law prohibits selling alcohol to adolescents under 18 years of age, most Brazilian adolescents have at least once in their lifetime purchased alcoholic beverages without being asked for identification.^26^ The *Política Nacional sobre o Álcool *(Brazilian Alcohol Policy) was launched in 2007,^h^ but the implementation of this policy is still largely disorganized. A clear focus of this policy has been on disaggregating the relationship between alcohol consumption and motor vehicle accidents by not combining drinking and driving. In 2008, the public countermeasures to reduce the alcohol-related burden were informed by two federal laws prohibiting the sale of alcoholic beverages on the highways and penalizing drivers caught with high levels of blood alcohol (Brazil, Decree 6117/2007).

The high prevalence of alcohol use among Brazilian students in all the cities studied points that many gaps remain to be filled for a comprehensive antialcohol policy. According to Laranjeira and Mistushiro,^18^ the unregulated market of alcohol in Brazil has contributed to the worsening health of the Brazilian population in the past decades, supporting the position of alcohol misuse as one of the major public health problems in Brazil. One of the hypothesis to explain the unregulated alcohol market, even considering the existence of some laws that aim to regulate alcohol sales and purchase, is that Brazil is home of one of the largest breweries in the world – *Companhia de Bebidas das Américas* (AMBEV) –, which has a strong political influence on the Country.^25^

The parallel decrease in the use of both substances, even without a robust antialcohol policy, suggests possible results of multidrug prevention efforts or the reduction in polydrug use by adolescents. In the USA, tobacco use in adolescents was associated with higher rates of other substance use disorders, particularly alcohol. Analysis of data from the 2007-2011 U.S. National Survey on Drug Use and Health, with a total of 91,152 household-dwelling adolescents (ages 12-17) randomly selected, showed that, when compared with nonusers of tobacco, users were nine times more likely to have alcohol use disorders.^5^ This is consistent with Gossop’s hypothesis^13^ that “few drug takers confine themselves to using a single substance. Cigarettes and alcohol often go together”. For instance, according to the present study, in 2010 Curitiba was the Brazilian city with the highest alcohol use prevalence and the only one showing increased tobacco prevalence in the 21-year trend. Our finding does not support the role of tobacco and alcohol as independent drug-related problems, suggesting more comprehensive approaches to prevent tobacco and alcohol misuse.

Overall, alcohol and tobacco use among adolescents has been declining in several countries, and causal effects from governmental prevention strategies are always mentioned.^16^ Therefore, we cannot ignore the actions of the Brazilian government for the decrease in drug demand, which may be indirectly contributing to alcohol and tobacco prevalence decrease among students. In 1998, the National Secretariat for Drug Policy (SENAD) was established under the Brazilian Ministry of Justice, which, among other roles, coordinates the national activities for prevention of drug abuse. Their main public action was the creation of several distance learning courses on drug prevention, aiming to train school teachers, health professionals, religious and community leaders in drug abuse prevention, reaching more than two million people in the period.

A substantial geoeconomic region difference in tobacco smoking and alcohol use prevalence suggests that SENAD public policies and actions are being implemented differently among cities and are greatly influenced by local cultural factors. The recent rise in smoking prevalence in Curitiba deserves in-depth investigation and calls for a more systematic approach to drug use in the high-risk group of adolescents. Universal prevention in the school setting is one of the most feasible and appropriate strategies to tackle substance use among youth.^10^ ^,^ ^28^ However, most Brazilian schools lack an ongoing project to prevent drug use nor undertake culturally adapted activities to face this problem.

Despite the reduction in legal drug use in Brazil in the past two decades, effective drug use prevention programs should be included in the school curriculum. According to a recent national survey, students attending public school begin to use alcohol at 12.5 years of age;^20^ thus, school-based prevention programs may be included starting at the elementary school curriculum.

The data of this study are based upon self-report; therefore, questions were subject to interpretation by the participant and to a possible information bias. The anonymous nature of the survey and the absence of the teacher in the classroom during the survey administration should help promote response validity. In addition, the question about a fictitious drug allowed us to drop the questionnaires with proved bias information. Further, we lacked data from private high schools students, so they are not generalizable to the overall adolescent population in Brazil, but only to those attending public schools in 10 Brazilian state capitals, who comprise 85.0% of the adolescent population in these cities.^i^

The strength of this study included using a standardized methodological procedure to diagnose and monitor alcohol and tobacco use among Brazilian adolescents living in all of the geoeconomic regions. Monitoring trends in adolescent alcohol and tobacco use over time is important for planning the allocation of resources and for evaluating the general impact of public policy for prevention.

In summary, we set out to monitor in surveys of standardized methodology the trend of past-year prevalence of smoking and alcohol use among Brazilian adolescents attending public school in the five Brazilian regions, from 1989 to 2010. Although the regions differ culturally, economically, socially, climatically and racially from each other, students living in all regions presented high alcohol use prevalence during the whole period analyzed.

The decrease in tobacco use prevalence detected all over the country can be related to the successful and comprehensive Brazilian antitobacco policies. Aspects of the local alcohol culture remain unchangeable in Brazil; however, public policies have apparently influenced reduction in consumption, although in much smaller proportions than those observed for tobacco.

## Footnotes

a Organización de los Estados Americanos. Observatorio de Seguridad Ciudadana: repositorio de datos. Washington (DC); c2015 [cited 2014 Sept 14]. Available from: http://www.oas.org/dsp/Observatorio/database/countries.aspx?lang=es

b Servicio Nacional para la Prevención y Rehabilitación del Consumo de Drogas y Alcohol. Décimo Estudio Nacional de Drogas en Población Escolar: principales resultados nacionales. Santiago: Observatorio Chileno de Drogas; 2014 [cited 2014 Sept 7]. Available from: http://www.senda.gob.cl/wp-content/uploads/2014/07/decimo-escolares-2013.pdf

c Inman P. Brazil overtakes UK as sixth-largest economy. *The Guardian *[Internet]. 2011 Dec 26 [cited 2014 Sept 1]. Available from: http://www.theguardian.com/business/2011/dec/26/brazil-overtakes-uk-economy

d Instituto Brasileiro de Geografia e Estatística. Censo demográfico 2010: características da população e dos domicílios. Rio de Janeiro (RJ): IBGE; 2011.

e Carlini-Cotrim B, Barbosa MTS. Pesquisas epidemiológicas sobre o uso de drogas entre estudantes: um manual de orientações gerais. São Paulo (SP): CEBRID/UNIFESP; 1993.

f Comissão Nacional para Implementação da Convenção Quadro para Controle do Tabaco. A convenção quadro para controle do tabaco (COP 5) e produção de fumo: análise de situação: sumário executivo; 2012 [cited 2014 Sept 1]. Available from: http://www2.inca.gov.br/wps/wcm/connect/ff11c6004eb68a33a0dfb2f11fae00ee/artigos_17_18_+cenario+atual.pdf?MOD=AJPERES&CACHEID=ff11c6004eb68a33a0dfb2f11fae00ee

g Carlini ELA, Noto AR, Sanchez ZM, Carlini CMA, Locatelli DP, Abeid LR, et al. VI Levantamento Nacional sobre o consumo de drogas psicotrópicas entre estudantes do ensino fundamental e médio das redes públicas e privadas de ensino das 27 capitais brasileiras, 2010. São Paulo (SP): CEBRID/UNIFESP; Brasília (DF): SENAD; 2010.

h Brasil. Decreto 6.117, de 22 de maio de 2007. Aprova a Política Nacional sobre o Álcool, dispõe sobre as medidas para redução do uso indevido de álcool e sua associação com a violência e criminalidade, e dá outras providências. *Diario Oficial Uniao* 2007; 22 maio

i Instituto Nacional de Estudos e Pesquisas Educacionais Anísio Teixeira. Escolas públicas atendem 45 milhões de alunos no Brasil. Brasília (DF): INEP; 2014 [cited 2014 Jan 1]. Available from: http://portal.inep.gov.br/rss_censo-escolar/-/asset_publisher/oV0H/content/id/19910
